# “You don't put it down to arthritis”: A qualitative study of the first symptoms recalled by individuals with knee osteoarthritis

**DOI:** 10.1016/j.ocarto.2023.100428

**Published:** 2023-12-16

**Authors:** L.K. King, A. Mahmoudian, E.J. Waugh, I. Stanaitis, M. Gomes, V. Hung, C. MacKay, J.W. Liew, Q. Wang, A. Turkiewicz, I.K. Haugen, C.T. Appleton, S. Lohmander, M. Englund, J. Runhaar, T. Neogi, G.A. Hawker, OARSI Early-stage Symptomatic Knee Osteoarthritis Initiative

**Affiliations:** a St. Michael's Hospital, Unity Health Toronto, Toronto, Canada; b Department of Medicine, University of Toronto, Toronto, Canada; c Department of Clinical Sciences Lund, Orthopedics, Clinical Epidemiology Unit, Lund University, Lund, Sweden; d Department of Movement Sciences and Health, University of West Florida, FL, USA; e Department of Physical Therapy, University of Toronto, Toronto, Canada; f Women's College Research Institute, Women's College Hospital, Toronto, Canada; g West Park Healthcare Centre, Toronto, Canada and Department of Physical Therapy, University of Toronto, Toronto, Canada; h Boston University Chobanian & Avedisian School of Medicine, Boston, MA, USA; i Department of Orthopedics, Shanghai Sixth People's Hospital, Shanghai, China; j Department of General Practice, Erasmus MC University Medical Center, Rotterdam, the Netherlands; k Center for Treatment of Rheumatic and Musculoskeletal Diseases (REMEDY), Diakonhjemmet Hospital, Oslo, Norway; l Department of Medicine and Department of Physiology and Pharmacology, Schulich School of Medicine and Dentistry, The University of Western Ontario, London, Canada; m Western Bone and Joint Institute, London, Canada; n Department of Clinical Sciences Lund, Orthopedics, Lund University, Lund, Sweden

**Keywords:** Knee osteoarthritis, Symptoms, Qualitative research, Patient-orientated research

## Abstract

**Objective:**

As part of the first phase of the OARSI Early-stage Symptomatic Knee Osteoarthritis (EsSKOA) initiative, we explored the first symptoms and experiences recalled by individuals with knee osteoarthritis (OA).

**Design:**

This qualitative study, informed by qualitative description, was a secondary analysis of focus groups (n ​= ​17 groups) and one-on-one interviews (n ​= ​3) conducted in 91 individuals living with knee OA as part of an international study to better understand the OA pain experience. In each focus group or interview, participants were asked to describe their first symptoms of knee OA. We inductively coded these transcripts and conducted thematic analysis.

**Results:**

Mean age of participants was 70 years (range 47–92) and 68 ​% were female. We developed four overarching themes: *Insidious and Episodic Onset*, *Diverse Early Symptoms, Must be Something Else*, and *Adjustments*. Participants described the gradual and intermittent way in which symptoms of knee OA developed over many years; many could not identify a specific starting point. Participants described diverse initial knee symptoms, including activity-exacerbated joint pain, stiffness and crepitus. Most participants dismissed early symptoms or rationalized their presence, employing various strategies to enable continued participation in recreational and daily activities. Few sought medical attention until physical functioning was demonstrably impacted.

**Conclusions:**

The earliest symptoms of knee OA are frequently insidious in onset, episodic and present long before individuals present to health professionals. These results highlight challenges to identifying people with knee OA early and support the development of specific classification criteria for EsSKOA to capture individuals at an early stage.

## 1 Introduction

The personal and societal impact of knee osteoarthritis (OA) is substantial and growing [1], yet there are no safe and effective medications approved by regulators to slow, halt or reverse its progression. To date, clinical trials of disease modifying OA drugs (DMOADs) and other potential therapies have mostly focused on those with established or late-stage OA [2], that is, those with definite radiographic features of OA, limited by lack of a standardized way to identify individuals with early-stage symptomatic knee OA (EsSKOA) [3]. To enroll participants into clinical trials of DMOADs and other disease modifying therapies before there is established radiographic structural change, the OARSI EsSKOA initiative seeks to develop classification criteria for EsSKOA. The first phase of this initiative involves incorporating the perspectives of diverse stakeholders, including people living with symptomatic knee OA, to enhance our understanding of EsSKOA, and inform candidate items that might help discriminate EsSKOA from other causes of knee symptoms and from established or late-stage knee OA.

An in-depth understanding of the first features of knee OA remains elusive, owing to the limited capacity to capture individuals early in the course of their illness and disease. Those with relevant lived experiences are best able to provide these insights. The few studies to date that have sought patient perspectives on first manifestations of knee OA have focused more on the evolution of symptoms over time. A single qualitative study involving 17 Taiwanese individuals aged 40–55 years explored the experiences recalled by people with knee OA at an early age between initial symptom onset and diagnosis of OA. Initial symptoms recalled included pain, joint stiffness, and crepitus that gradually increased in frequency and intensity and that led patients to seek care to confirm a diagnosis [4]. The change in the pain experience over the course of living with knee OA, beginning typically with intermittent pain, is well described [5], but less is understood about other OA signs and symptoms.

To enhance our understanding of EsSKOA, we comprehensively explored the first symptoms and experiences of knee OA recalled by individuals with knee OA, using qualitative methods.

## 2 Methods

### 2.1 Study design

This qualitative study involved a secondary analysis of focus groups and one-on-one interviews that were completed in 2006 as part of an OMERACT/OARSI initiative to better understand the OA pain experience and changes in pain over time [5].

The analysis was informed by the methodology of qualitative description. Qualitative description seeks to enable a comprehensive account of experiences [6], staying close to the data with low level of interpretation [7], and is a relevant approach in studies that seek to acquire information from those experiencing the phenomenon under investigation within health sciences [8]. Its philosophical underpinnings is that it is an inductive process, to develop, understand and describe phenomena, and in keeping with an interpretivist stance, the researcher is active in the research process [8].

All participants provided written informed consent, and this study was approved by the Women's College Hospital research ethics board (REB# 2022-0098-E). We followed the Consolidated Criteria for Reporting Qualitative Studies (COREQ) guidelines for reporting qualitative research.

### 2.2 Sampling and data collection

We analyzed transcripts of focus groups (n ​= ​17 groups) and one-on-one interviews (n ​= ​3) conducted with a total of 91 individuals living with symptomatic knee OA [5]. Focus groups and interviews with individuals with hip OA as the index joint were not included. Focus groups and interviews were completed at five centres: Australia (Sydney), Canada (Toronto and Vancouver), UK (Bristol), and USA (Houston), and participants were organized by pain severity on 10-point numeric rating scale (“mild [1–4] to moderate [5–7]” and “moderate [5–7] to severe [8–10]”). Participants were recruited from the community via advertisements, clinical practices of the investigators, and from investigators’ research cohorts, and were purposively sampled for a range of disease duration and symptom severity [5].

To be eligible, participants had to speak English, be ​≥ ​40 years, and responded “yes” to: ‘‘Have you experienced aching, discomfort, pain and/or stiffness in or around a knee on most days of at least 1 month (15 or more days of the month) during the past year?’‘. To confirm diagnosis of OA, these individuals then underwent xray of the symptomatic knee (positive if OARSI grade 1 or greater for any of: joint space narrowing, osteophytes, sclerosis, or subchondral cysts [9]). Anyone who had experienced an injury to the joint area within the last year, received a joint replacement of the symptomatic joint, or with concurrent rheumatoid arthritis or any other type of inflammatory arthritis, fibromyalgia, chronic low back pain, or another chronic pain disorder was excluded.

The format of the focus groups was standardized across centres, and each session followed a structured interview guide. One-on-one interviews were also conducted in Toronto using the same interview guide. Focus groups, comprised of up to eight participants, were approximately 2.5 ​h in length, and led by a local trained moderator. Each focus group or one-on-one interview used a ‘‘funnel approach’‘, starting with broad open-ended questions followed by increasingly focused questions. The interview guide included questions to elucidate detailed descriptions of experienced knee pain or other sensations and explore the changes in these characteristics over time. Relevant to our research question, participants were asked to describe their first symptoms of knee OA: “*Not everybody with knee problems describes pain as the first sensation. What were the first sensations or symptoms for you that you felt in your knee(s)?”* Probing questions included: *How frequent were these symptoms? How intense were these symptoms? How long did these symptoms last? Did you use pain medication or other treatments at the beginning?* Full interview guide is shown in Appendix 1.

To characterize the sample, at the end of each focus group or interview, participants completed a brief questionnaire assessing sociodemographic characteristics (age, sex, level of education, racial/ethnic background), the self-reported duration of knee symptoms *(“How long has it been since you first noticed your knee?“*), and knee OA symptom severity using multiple measures (Western Ontario and McMaster University Osteoarthritis Index [WOMAC], Knee Injury and Osteoarthritis Outcome Score [KOOS], and a 10-point numeric rating scale [NRS] for pain severity over the past 3 months).

Interviews were digitally recorded and transcribed verbatim by a professional transcriptionist. We organized the data using a qualitative software program, NVivo 12 (QSR International Pty Ltd).

### 2.3 Analysis

We conducted inductive thematic analysis informed by Braun and Clarke [10]. Two researchers (LK and MG) became familiar with the data and met regularly to discuss their interpretations. The first author inductively developed an initial coding framework. Our analytic team was composed of six researchers (LK, EW, AM, IS, VH, MG), including one rheumatologist with experience in conducting qualitative research, two physical therapists and two research assistants with experience with OA research, and one summer research student. All members of the analytic team line-by-line coded four transcripts independently and then met to compare codes and propose new codes. Through these meetings we revised the coding framework. Once the coding framework was established, with agreement on how to apply codes, we divided the remaining 16 transcripts among members of the analytic team to code in duplicate, with discrepancies in coding resolved by consensus.

In an iterative and reflective process, the codes were then organized into groups, and we identified themes based on repeated patterns across the data relevant to the research aims. Memos were written to support development of themes. Constant comparative analysis allowed in-depth insights into similarities and differences across transcripts [11]. We reviewed themes and supporting data with a large research team with methodological and substantive expertise (CM, JL, QW, AT, IH, JR, TA, ME, SL, TN, GH) and kept an interrogative stance towards the data [12]. We applied the eight key markers of rigour in qualitative research, as described by Tracy [13]. This included use of multiple analysts, analytic memos, an audit trail and supporting data that provided rich rigour, use of thick description, which enhanced credibility and we used reflexive research practices [13].

## 3 Results

We included focus group and interview data from 91 participants (detailed characteristics in Table 1). The median age of participants was 70 years (range 47–92), 68.1 ​% were female, median WOMAC pain was 8.9/20 (range 0–17), and the median self-reported duration of knee joint symptoms was 6 years (range 0–54).

**Table 1:** Characteristics of focus group and interview participants.

	n ​= ​91
Age in years, median (min-max)	70.0 (47–92)
Female, n (%)	62 (68.1)
Caucasian, n (%)	88 (96.7)
Education, n (%)	
< ​High school	12/77 (15.6)
Completed high school	12/77 (15.6)
≥ ​Post-secondary	53/77 (68.8)
Duration of OA in years, median (min-max) (n ​= ​76)	6.0 (0–54)
NRS pain severity (1–10), median (min-max) (n ​= ​77)	5.5 (1–10)
WOMAC^a^, median (min-max) (n ​= ​90)	
Total (0–96)	45.5 (0–75.3)
Pain (0–20)	8.9 (0–17)
Function (0–68)	29.7 (0–55.3)
Stiffness (0–8)	4.8 (0–7)
KOOS^b^ subscales, median (min-max)	
Pain (n ​= ​90)	74.5 (22.2–100.0)
Symptoms (n ​= ​90)	73.2 (25.0–100.0)
ADL (n ​= ​89)	74.1 (20.6–100.0)
Sports/Rec (n ​= ​86)	54.1 (0.0–100.0)
Quality of Life (n ​= ​90)	57.9 (0.0–100.0)

a For worst knee.

b For worst knee, possible range: 0 (extreme problems) – 100 (no problems).

From the data, we developed four overarching themes: *Insidious Onset*, *Early Symptoms, Must be Something Else*, and *Adjustments*. We present our findings with illustrative quotes from the focus groups and interviews, with additional quotes presented in Table 2. We denote focus groups (FG) by the site and participant group (e.g., FG Canada, Toronto 1).

**Table 2:** Themes with additional illustrative quotes.

Theme	Illustrative Quotes
Insidious and Episodic Onset	*“I think initially, ahm, a lot of it started in an insidious way in that, ah, oh, you go to bed and your leg would be tired or it would be sore and achey, and you wouldn't think too much of it …” -* FG Canada, Toronto 2 *“… it doesn't sort of suddenly come, you know, it's a gradual thing.” -* FG UK 3 *“… gradually more and more.” -* FG Canada, Toronto 1 *“It was like it would come on and then disappear, and come on again. I'd be in the shopping mall, I feel this, ah, just pain in my legs. And it continued like that.”* - FG USA 3 *“Intensity was not at much at first ….”* - FG Canada, Toronto 1 *“It started off just with a sort of little needle in my … and it got worse and worse for longer and longer and longer periods.”* - FG Australia 3
Diverse Early Symptoms	*“Mine started out when I was active, and that's when my knees would hurt, originally.”* - FG Canada, Toronto 1 *“Yeah, I don't think it even occurred to me that I had a problem, except [when] I did stairs.”* - FG Canada, Vancouver 1 *“… no pain, only stiffness.”* - FG Australia 3 *“you get crunching”* - FG UK 3 *“But, the one thing I can remember is the (unclear), used to be a clicking noise.”* - FG Canada, Toronto 1 *“… [challenges in] getting up from the floor (laughs).”* - FG USA 2
Must Be Something Else	*“I've always done a lot of walking, and I think ahm, also fallen on my knees a lot, so I think at first I just thought it was a bruise.” -* FG Canada, Toronto 3 *“maybe I hurt myself in the garden.” -* FG Canada, Toronto 4 *“Yeah all the concrete that's put down, and then the pressure on the knees because of that?”* - FG UK 3 *“I couldn't believe it was arthritis.”* - FG Canada, Toronto 5
Adjustments	*“I didn't give up walking, and I just stayed more or less on a more level [ground].” -* FG Canada, Vancouver 1 *“But it's just ahm, I guess just not being able to run. (Laughter) That gets your attention.”* - FG USA 2 *“Can't drive long distances.”* - FG Australia 1

### 3.1 Theme 1: Insidious and Episodic Onset

Participants consistently described the gradual way symptoms of knee OA had developed, usually over many years.

*“But it came, basically, gradually.”* - FG Canada, Vancouver 2

*“Ah, I'd say it was a gradual deterioration, a gradual growth.” -* FG USA 2

For most, the onset was so insidious, they could not identify a specific starting point for their OA illness. Participants related this to the low intensity of symptoms at the onset that they generally perceived to be innocuous in the moment.

*“I guess like the others I can't identify specifically when my symptoms started.”* - FG Canada, Vancouver 3

*“Started off as soreness and you stopped thinking about it.”* - FG Australia 1

Participants described an intermittent, episodic, nature to their earliest symptoms. Often periods of symptoms would be interspersed with long periods with minimal or no symptoms. Each time symptoms waned, this was interpreted as resolution of the problem. Only with the benefit of hindsight could participants link recurrent episodes of noticing their knee symptoms as the start of their OA illness.

*“Off and on it would ache.”* - FG Canada, Toronto 1

*“After that first go-around it was gone. It was gone! I did not have any knee pain for a long, long time. Then I began to get a little twinge here and a little twinge there. It must have been six or eight months before I even started getting these twinges, or discomfort, this mild discomfort.”* - FG USA 2

Some individuals who recalled a joint injury found it challenging to recall their earliest OA symptoms because of difficulty separating onset of OA with symptoms related to their initial injury. For example,

*“No I can't tell you [about the onset of OA symptoms] because I guess it's the first ahm, the first … at first I couldn't separate out little things from the original injury that I had when I broke a ligament.”* - FG Canada, Vancouver 2

### 3.2 Theme 2: Diverse Early Symptoms

Participants recalled diverse initial knee symptoms, enumerating a number of different manifestations. Frequently reported early symptoms were activity-related knee pain, joint stiffness and crepitus.

Participants described noticing knee pain only during weight-bearing or high-impact activities such as walking, running or climbing stairs.

*“ … doing stairs, that's when I first noticed it.”* - FG Canada, Toronto 3

*“[Pain with] walking or dancing or doing some exercises.”* - FG Australia 3

Many participants spoke about joint stiffness as being one of their initial joint symptoms, and for some it was their sole initial manifestation.

*“… at first I never noticed anything except for the stiffness, the mobility [of the knee].”* - FG Canada, Toronto 2

*“I've never had a lot of pain, I had more stiffness when it first started.”* - FG Canada, Toronto 3

*“Just the stiffness round there, that’s all.”* - FG UK 3

Many participants described noting knee joint crepitus early in their illness.

*“I just had that clicking, that sound”* - FG Canada, Toronto 5

*“But it's not just the pain, it's the crunches. As you walk you hear I guess bone on bone or whatever crunches in there.”* - FG USA 1

Because of the insidious onset and the often years to decades that had passed since symptom onset, several participants struggled with specifying first symptoms. There were several participants who described that it was not the presence of a new symptom that they noticed, but rather a newfound inability to perform a physical function or activity.

*“And I'm trying to think of when I really–-I mean I noticed that my knees were aching, but I can't really put it to exactly when it started. And I found that I couldn't do some things that I--like kneel down, just to kneel, squat down. I used to do that with kids, I cannot--I can't do that.”* - FG Canada, Toronto 4

*“Just limited mobility in moving quickly and easily.”* - Individual Y133702

There was heterogeneity in the specific first symptoms recalled. Across participants, there was a range of initial manifestations described, from perceived muscle weakness, to locking, to sharp pain, to joint swelling.

*“My knees were swollen …” -* FG Canada, Vancouver 1

*“… just seemed to lock.” -* FG Canada, Toronto 4

*“Ahm, it was more of a weakness in the knees.” -* FG Canada, Toronto 2

*“Ahm, pain, sharp. Sharp, shooting pain.*” *-* FG Canada, Toronto 3

### 3.3 Theme 3: Must Be Something Else

Many participants described how they initially rationalized the presence of their knee symptoms, minimizing any initial reason for alarm. They would attribute their symptoms to an injury, even if they could not recall a specific event occurring, to pre-existing conditions they had, or even to “getting old”.

*“I didn't imagine it was arthritis, I thought, well I must have done something to it, but I couldn't remember what.”* - FG Canada, Toronto 5

*“… because you’re getting older and you’ve got all these aches and pains.”* – FG Canada, Vancouver 1

*“I would guess that when you're involved in sports like that on a regular basis, that you're just going to say, 'I'm just sore from the tennis game yesterday or the softball game. And so you dismiss it and don't really think of it as arthritis pain to begin with.”* - FG USA 2

Several participants described how they did not allow themselves to believe their symptoms were due to arthritis since that would mean facing that they were living with a chronic condition.

*“Well, because at first you're in denial and you don't--I mean I didn't think it was arthritis.”* - FG Canada, Vancouver 1

*“And I would never want to believe I had arthritis.”* - FG Canada, Toronto 2

### 3.4 Theme 4: Adjustments

Participants described how they engaged in adaptive strategies to allow them to continue participating in recreational or other daily activities, for example by incorporating more rest or shifting activity type. These adjustments would allow them to continue an essentially normal daily life, and maintain the perception that there was no impact on level of activity.

*“I had to just curtail certain sports … I went from like skiing five hours to two-hour increments, taking a break, because the knee would just collapse and I'd fall. So it stopped. And I just adjusted. I didn't stop doing anything, but I just changed my time frames and just built in more rest periods which I hadn't done before.”* - FG Canada, Vancouver 3

*“So I guess I wasn't thinking arthritis, but it hurt to jog so I started walking, and that didn't seem to be a problem.”* - FG USA 3

*“When you first feel the pain, you are still walking the way you expect to walk and so it's very painful. And then you learn to adapt. So you learn to work with it and gradually the pain kind of doesn't seem as severe because you're not getting up as often, you're pampering yourself a little bit, you're being more careful.”* - FG Canada, Toronto 5

For others, their initial actions included suppressing awareness of symptoms to enable them to continue on with their usual activities as best they could.

*“I'd have a little pain now and then but just tried to ignore it” -* FG Australia 3

*“Probably that's why you don't put it down to arthritis because you're just putting up with pain because you've got something to do, you want to get that done.”* - FG Canada, Toronto 1

Participants reported placing greater importance on their knee symptoms only when functional impairment from knee-related symptoms limited their ability to perform valued activities. It was then that they were more likely to seek and engage in care.

*“I first noticed it really when I’ve had to give up golf – that’s walking, you know, I found that I couldn’t keep up, and I had to pack it in.”* - FG UK 3

*“Well [began to engage in OA care when] it was bad enough that I wasn't able to go for walks with the grandkids and that sort of thing, and it was interfering with what I wanted to do in my life.”* - FG Canada, Vancouver 3

It was limitations in valued activities, and no longer being able to easily compensate and adapt in light of their joint symptoms, that often provided the signal that something was wrong. It generally occurred years after symptom onset.

## 4 Discussion

This international qualitative study illuminates the earliest recalled symptoms and experiences of people living with knee OA. Initial symptoms that developed slowly, often over many years, were insidious in onset, intermittent, of low intensity, and varied in type and nature. Participants generally did not attribute them to arthritis in the moment. Individuals countered these symptoms with adaptive strategies to facilitate, at least initially, continuing to participate in both daily and recreational activities. For many, their symptoms became top-of-mind, and there was impetus to seek care, only when they became more persistent and resulted in loss of function. These results importantly expand our current understanding of the signs and symptoms of knee OA, and individuals’ perceptions of the symptoms, and highlight the current challenges to identifying people with knee OA early in its course.

A key finding from this study was the clear description from most participants of their knee OA symptoms starting as a slow, insidious process, creeping up on them over years. Participants’ lack of initial alarm to their symptoms reflected the long time they had to make gradual adjustments in their lives to compensate, keeping awareness of an overt change in their health suppressed. Prior qualitative studies in people with OA have described the use of strategies to mitigate impact of symptoms and disability through change in behaviours and compensatory adaptations [14]. These adjustments, including “being careful” [15], or changing how activities are performed [16], may permit individuals with knee OA to manage for some time perceiving their symptoms are unchanged [17]. Given the perceived low threat of initial OA symptoms, participants delayed care in keeping with the Health Belief Model [18], where the perception of personal threat of illness predicts the likelihood a person will engage in individual health behaviours such as seeking treatment. This underscores a potential challenge of the EsSKOA initiative whereby we hope to develop classification criteria to identify individuals sufficiently early, when there might be an opportunity to change illness and disease evolution [2], while also at a juncture where individuals are willing to accept treatment, including participating in OA trials.

Results from this study highlight current challenges in identifying people with knee OA at an early stage. First, participants frequently recalled an episodic nature to their initial symptoms, where periods of experiencing symptoms could be interspersed by prolonged periods of being asymptomatic. Therefore, individuals who are asymptomatic in the moment may still be experiencing the illness of knee OA though may not be picked up by epidemiologic definitions of OA nor typical trial inclusion criteria. Prior research has suggested that people with knee OA often struggle to understand the meaning of intermittent symptoms at the time [15] which may explain the reluctance that participants had initially believing their symptoms could be due to OA. Second, there was wide variability in the initial knee symptoms recalled by our study participants, which may be related to the heterogeneous pathogenesis of knee OA [[19], [20], [21]]. It is possible that different etiologies, e.g., stemming from risk factors of knee injury versus obesity, are associated with different patterns of initial manifestations. If so, a “one size fits all” approach to classification criteria may not be appropriate and could require considering incorporating different variables in a composite manner [22]. Third, we found that stiffness was frequently reported as an important early symptom and could be prolonged. This finding is inconsistent with the 1986 ACR classification criteria for knee OA [23], which stipulate “less than 30 ​min of morning stiffness”, and the NICE criteria for OA that include “either no morning joint-related stiffness or morning stiffness that lasts no longer than 30 ​min” [24]. Ongoing work will need to clarify the discriminant validity of duration of morning stiffness in identifying our target population, EsSKOA, from other conditions causing knee symptoms.

Following ACR-EULAR methodology [25], the first phase of developing classification criteria includes item generation, where it is important to include patient perspectives. We had initially conceptualized this study as a content analysis [26] of focus groups and interview transcripts to compile a list of the first symptoms of knee OA as described by patients that could be incorporated with other stakeholder perspectives and inform candidate items. After initial readings of the transcripts, it became clear that participants found it challenging to enumerate their initial symptoms, define them, and describe the context in which they occurred. We have chosen to illuminate these perspectives in this qualitative description study that adds to the paucity of research in this area and forms the basis for further work. The present study has described these challenges recalling initial manifestations owing to the slow, insidious way in which OA develops, dismissal of initial symptoms, and initial compensatory mechanisms, and has served to importantly inform a greater understanding of the earliest experiences of living with OA.

Our study has a number of strengths. We included diverse perspectives of people living with knee OA, leveraging an already-collected focus group and interview data from participants from four countries and three continents who were purposively sampled for variation in age and symptom severity, in addition to balance of male/female representation. This has allowed us to capture broad illness experiences, including variation in initial manifestations. Most of the data were drawn from focus groups, an ideal method for exploring individuals’ own meanings and understandings of their illness, with the group dynamic often facilitating openness and disclosure [27]. We perceived there was thematic saturation within our data [28]. We had a large analytic team that included individuals with different professional roles and thus a diversity of perspectives, exploring alternating explanations, and an overall interrogative stance to the data promoting rigour [12]. Our study also has a number of limitations. Many of our study participants had a long disease duration with described difficulty recalling initial symptoms that for some began several decades earlier, limiting amount and potentially accuracy of data recalled. While participants were asked to describe their first symptoms, they were not provided a time frame to think about (e.g., within the first year of first noticing their knee). Therefore, the meaning of “first” may have been interpreted differently across participants, potentially including perspectives of symptoms many years into the illness. While data were collected in 2006, societal attitudes towards OA, and management strategies, are largely unchanged. There are limitations inherent in a secondary analysis, including that data collection and analysis could not occur concurrently in an iterative process [29], inability to adapt interview guides, nor perform member checking. Further prospective research incorporating patient perspectives may overcome some of these limitations. Finally, earliest symptoms, as described by participants, cannot necessarily be equated to the earliest stages of knee OA versus another cause of symptoms, but the themes provide a framework of symptoms to consider and further examine.

In conclusion, this study illuminates important patient perspectives on the first symptoms and experiences of knee OA, an area that has been under-researched. It furthers our understanding of knee OA symptoms as developing slowly, insidiously, and episodically. As a result, individuals with early symptoms tend to blame their symptoms on other factors, such as over-exertion, and generally do not seek care until functional limitations occur. This study provides important background context to further work to incorporate patient perspectives into the generation of candidate items for classification of EsSKOA.

## Role of the funding source

The sponsors had no role in the design and conduct of the study; collection, management, analysis, and interpretation of the data; preparation, review, or approval of the manuscript; and decision to submit the manuscript for publication.

## CRediT authorship contribution statement

All authors have contributed to the following: (1) the conception and design of the study, acquisition of data, or analysis and interpretation of data; (2) drafting the article or revising it critically for important intellectual content; and (3) final approval of the version to be submitted.

Lauren K. King and Gillian A. Hawker take responsibility for the integrity of the work as a whole, from inception to finished article.

## Conflict of interest

LKK reports support from Canadian Institutes of Health Research Doctoral Award, and grant funding from Physician Services Incorporated (PSI) Resident Grant; JWL reports a Pfizer research grant completed in 2021, unrelated to this work; QW reports support from Osteoarthritis and Cartilage as an Associate Editor for statistics; AT reports support from Osteoarthritis and Cartilage as an Associate Editor for statistics, IKH reports consulting fees from AbbVie, GSK and Grünenthal, and leadership role as the OARSI Board of Directors Secretary; CTA reports grant funding and consulting/honoraria from AbbVie, Novartis, Pfizer, and Servier; LSL reports consulting/honoraria from Arthro Therapeutics AB, Sweden, and participation on a DSMB board for AstraZeneca; ME reports grant funding from Swedish Research Council, ERC, ALF, Kock Foundations, Gustav V 80 ​Year ​B-day foundation, NORDFORSK, FOREUM, Foundation for support to people with movement disability in Skåne, Österlund Foundation, SUS donations, consulting from Cellcolabs AB, and leadership role as the OARSI President-elect; JR reports a leadership role on the OARSI Ethics & Governance Committee, Nominating Committee; and TN reports grant funding and consulting/honoraria from Pfizer/Lilly, Regeneron and Novartis. All other authors report no competing interests.
