# Supplementary material for: “You don't put it down to arthritis”: A qualitative study of the first symptoms recalled by individuals with knee osteoarthritis
Source: Osteoarthr Cartil Open. 2023 Dec 16;6(1):100428. doi: 10.1016/j.ocarto.2023.100428 (PMC10790080; doi:10.1016/j.ocarto.2023.100428)
Supplement: Multimedia component 1 [file mmc1.docx]

**Appendix 1 – Interview Guide**

**Introductions / Welcome**

**Overview of the objectives** of the focus groups - The purpose of today’s focus group is to gain a better understanding of the natural history of the arthritis pain experience - that is, how the pain and other sensations experienced by people with knee OA change over time as their arthritis progresses from the first twinge felt to the present time.

Our interest today is specifically on pain and other sensations in the knee, and the impact of the pain on your life. Another group is focusing specifically on the physical limitations people experience due to their arthritis. Today, we will be discussing your physical activities only in as much as your knee pain and other symptoms prevent you from doing these activities.

We hope to use the information that we derive through these focus groups to create a series of brief “scenarios” that describe the typical person with OA from the very beginning to late in the disease so that doctors and patients alike might use these descriptors to gauge whether the OA has worsened, improved with treatment, etc.

There will be two distinct parts to the discussion today. The first part will focus specifically on the pain and other sensations that your knee arthritis causes you. The second part will focus on the impact your pain has had on your level of activity and overall quality of life.

**PART 1 – KINDS OF PAIN/SYMPTOMS and TIMELINE**

**Now we’d like you to think about the different kinds of pain or other sensations you experience.**

**Q1:**  **Is your knee pain always the same or do you experience different kinds of pain?**

- Can you tell us about your different kinds of knee pain?
- For example, is the pain you get when you walk or climb stairs different from the pain you might have when you’re sitting?
- How?

**Summary – different triggers to pain -- are there any more?**

- What are they?

**Q2:** **(Link)** **What kind of pain came first?**

- e.g. Night versus day pain? Are they different? What came first?
- e.g. Rest versus activity pain? Are they different? What came first?
- e.g. Night versus rest pain?

**Q3: Not everybody with knee problems describes pain as the first sensation. What were the first sensations or symptoms for you that you felt in your knee(s)?**

- How frequent were these symptoms?
- How intense were these symptoms?
- How long did these symptoms last?
- Did you use pain medication or other treatments at the beginning?

**Q4: Thinking about how your pain or sensation has changed over time can you describe those changes?**

- How it felt (sensation – e.g. throbbing)?
- Has it gotten more/less intense?
- More or less often?
- Longer lasting each time?
- Can you predict when the pain will come?
- Any new symptoms that happened later on but did not happen at the start?
- When did you start using medicine?
- Other treatments?
- Walking aids (e.g. sticks)

*If they comment that “the pain got worse” or “the pain became more severe” ask them to be specific about what they mean by “it became worse” or “more severe”.*

**Q5:** **Can you think back to when your pain/sensation first became a regular occurrence. How many months or years *after* it first started did that happen?**

**Q6: Have any of you experienced an improvement in your knee pain?**

- Can you tell me in what ways the pain is better now than before?
- Do you have any ideas about why your pain got better?

**PART 2 – IMPACT OF PAIN ON LIFE**

**In this next part, I’d like to talk about how living with knee pain has affected your life.**

**Q7:** **Which activities were first to be affected by your knee(s)?**

- Which ones stopped? (first, second, third)
- Which ones modified? (first, second, third)

Can you think back to when this happened … **How many months or years after the pain/sensation first started did that happen?**

**Q8: Are there other aspects of your life that your arthritis pain has affected? Tell me about them.**

**END**
